# Wireless MRI‐Powered Reversible Orientation‐Locking Capsule Robot

**DOI:** 10.1002/advs.202100463

**Published:** 2021-05-03

**Authors:** Onder Erin, Mustafa Boyvat, Jelena Lazovic, Mehmet Efe Tiryaki, Metin Sitti

**Affiliations:** ^1^ Department of Physical Intelligence Max Planck Institute for Intelligent Systems Stuttgart 70569 Germany; ^2^ Department of Mechanical Engineering Carnegie Mellon University Pittsburgh PA 15213 USA; ^3^ Institute for Biomedical Engineering ETH Zurich Zurich 8092 Switzerland; ^4^ School of Medicine and College of Engineering Koç University Istanbul 34450 Turkey

**Keywords:** magnetic actuation, magnetic resonance imaging, medical robots, millirobots, minimally invasive medical devices

## Abstract

Magnetic resonance imaging (MRI) scanners do not provide only high‐resolution medical imaging but also magnetic robot actuation and tracking. However, the rotational motion capabilities of MRI‐powered wireless magnetic capsule‐type robots have been limited due to the very high axial magnetic field inside the MRI scanner. Medical functionalities of such robots also remain a challenge due to the miniature robot designs. Therefore, a wireless capsule‐type reversible orientation‐locking robot (REVOLBOT) is proposed that has decoupled translational motion and planar orientation change capability by locking and unlocking the rotation of a spherical ferrous bead inside the robot on demand. Such an on‐demand locking/unlocking mechanism is achieved by a phase‐changing wax material in which the ferrous bead is embedded inside. Controlled and on‐demand hyperthermia and drug delivery using wireless power transfer‐based Joule heating induced by external alternating magnetic fields are the additional features of this robot. The experimental feasibility of the REVOLBOT prototype with steerable navigation, medical function, and MRI tracking capabilities with an 1.33 Hz scan rate is demonstrated inside a preclinical 7T small‐animal MRI scanner. The proposed robot has the potential for future clinical use in teleoperated minimally invasive treatment procedures with hyperthermia and drug delivery capabilities while being wirelessly powered and monitored inside MRI scanners.

## Graphical abstract

Rotation is coupled with translation for untethered magnetic resonance imaging (MRI)‐powered robots with a freely rotating magnet. To remove such a coupling issue, an orientation‐locking mechanism via a phase‐changing wax material is proposed. Wireless power transfer‐based heating is used to trigger the mechanism. This wireless heating also enables to trigger drug release and induce hyperthermia towards future potential therapeutic functionalities.

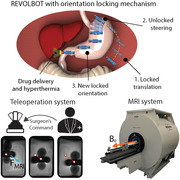

## 1 Introduction

Interventional magnetic resonance imaging (iMRI) provides non‐invasive imaging feedback in order to allow surgeons to monitor and perform minimally invasive interventions targeted inside the soft tissues of the human body, where a direct line‐of‐sight is unavailable. iMRI systems do not only acquire 3D anatomical images with non‐ionizing methods for guiding the surgeries, but also can provide further physiological information, such as perfusion, diffusion, blood oxygenation and thermometry.^[^ ^1^ ^]^ iMRI has been a clinical technology for a few decades and its hardware differs from the typical magnetic resonance imaging (MRI) hardware. It is typically an open bore system to allow a surgeon to have some access to the surgical region. Such open bore magnets have lower MR image resolution, sensitivity, and temporal efficiency.^[^ ^2^ ^]^ Moreover, the limited bore size may restrict the working space of the surgeon. In order to reduce such limitations, interventional magnetic resonance imaging systems (IMRIS) have been developed as a recent biomedical technology.^[^ ^3^ ^]^ The major advantage of such systems is utilizing a clinical, high‐resolution MR scanner, while allowing a large work space for the surgeons. IMRIS has a mobile clinical MRI device, i.e., can move inside the operation room (OR), when the operator needs to use an MR image feedback. Even though the patient is stationary on the bed, many of these procedures require the large MRI device to go in‐and‐out to acquire an MR image for guidance. This procedure may prolong the operation duration and sometimes create registration issues due to the motions during each iteration inside the bore. More importantly, since translating the large MRI device takes tens of seconds, it introduces a delay in case of any emergency, such as extubation, while the patient is being scanned inside the bore.^[^ ^4^ ^]^

In order to eliminate most of the above issues and to improve interventional robotic MRI procedures, MRI‐powered untethered robots may serve as a strong alternative. The principle of this method is using the magnetic field gradients already available inside clinical MRI devices to actuate remote magnetic materials while also monitoring them with the same MRI device. This method does not require iterative in‐and‐out procedures and has the advantage of direct monitoring of the operation continuously while performing teleoperation with these MRI‐powered robots. This image feedback information provided by MRI devices combined with MRI‐powering could assist clinical surgeries and pave the way for autonomous robotic operations.^[^ ^5^ ^]^ The initial studies of this concept have been explored in MRI‐based actuation, precise position control, and design and actuation of tethered medical devices (e.g., catheters) for various medical operations.^[^ ^6^, ^7^, ^8^, ^9^, ^10^, ^11^, ^12^, ^13^, ^14^, ^15^, ^16^, ^17^, ^18^, ^19^, ^20^, ^21^, ^22^, ^23^, ^24^ ^]^

There are three types of electromagnets inside MRI devices. These electromagnets are the main magnet, gradient coils, and RF excitation coil. In terms of actuation, the main magnet and the gradient coils have a key role. The main magnet is responsible to generate a very strong and unidirectional magnetic field along the bore of the device (i.e., 1.5–7 T). This magnetic field cannot be turned off or its intensity cannot be modulated. Such a magnetic field aligns every magnetic object under MRI, and also magnetizes every individual magnetic part along its direction. It is therefore very challenging to accomplish complex motions, such as undulation, spinning, or rolling, using magnetic field‐based torques. Unlike the custom electromagnetic actuation systems, where a simple temporal change of the magnetic field direction can induce a rotation on magnetic robots, the robot's orientation change inside the MRI is challenging due to the constraint imposed by the unidirectional main magnetic field. The actuation force on the magnetic objects, however, can be generated via magnetic gradient coil units. A magnetic object with a fixed body orientation can therefore translate while preserving its orientation thanks to the magnetic pulling forces exerted by the MRI gradient coils.

In respect of wireless MRI‐powered devices, previous studies present translational motion control of magnetic spherical particles towards tissue penetration, drug delivery, and embolization at various size scales.^[^ ^25^, ^26^, ^27^, ^28^, ^29^, ^30^, ^31^ ^]^ Such translational motion has been a major focus to have an autonomous position control with MRI feedback. Tracking and path planning with nonlinear model‐based approaches have shown the controllability of particles in planar surfaces and mazes.^[^ ^9^, ^11^ ^]^ To expand the system limitations, simultaneous imaging and actuation methods have been developed to increase the closed‐loop feedback rate towards real‐time tracking and control.^[^ ^7^, ^12^ ^]^ Multiple robot control^[^ ^6^ ^]^ and 3D motion control of these magnetic robots under pulsatile fluid flow conditions have also been demonstrated.^[^ ^32^ ^]^

In all of these studies, the navigation of such particles has been limited to mostly point‐to‐point position control. However, asymmetrically shaped capsule‐type miniature robots could require orientation control to steer them through tight regions and implement their medical functionality. To enable such orientation control capability for MRI‐powered capsule robots, Erin et al. have introduced a free‐to‐rotate spherical magnetic bead inside a capsule robot using MRI magnetic gradients, i.e., pulling forces.^[^ ^33^, ^34^, ^35^ ^]^ However, while having such orientation control capability, the robot could not have pure translational motion in 3D. Moreover, the magnetic pulling force effort to control the orientation simultaneously translated the robot, which resulted in a complex coupled motion. Such coupled motion in translation and rotation makes accomplishing a desired position with a desired orientation challenging for both open‐loop and closed‐loop control. Autonomous path planning and control methods were limited, demanding high feedback rates, and more importantly, requiring a large space to maneuver, which may not be available in many medical applications. Therefore, in this study, to separate 3D position control and planar orientation steering of such capsule robots independently and to reduce the need for large spaces for maneuvering, we propose a remote heating‐based reversible orientation‐locking robot (REVOLBOT), where the free‐to‐rotate spherical magnetic bead is embedded inside a phase (solid/liquid)‐changing wax material. Thus, the bead is unlocked and free to rotate when the wax is liquidized by remote heating, and it is locked in a given orientation when the wax is back to its solid phase at the body temperature. Such orientation locking mechanism enables orientation‐dependent steering in tight and narrow spaces, and simplifies the control of these untethered robots in medical tasks, which require various different poses of the robot by separating the underactuated motion into basic motion primitives. We envision the use of such a mechanism to navigate the robot in constrained spaces of peritoneal cavities, or inside of the stomach. Moreover, if there is a multiple target region, i.e., multiple tumor areas, sequential orientation‐locking and ‐unlocking may benefit surgeons to have an auxiliary freedom to guide such inherently underactuated robot dynamics with ease.

Various locking and unlocking mechanisms presented in the literature that were designed for tethered catheters for stiffness tuning or making the catheter insertion easier.^[^ ^36^, ^37^ ^]^ Due to their mechanism and steering functions, these mechanisms would not work properly under the magnetic conditions of MRI devices. Moreover, since these mechanisms are not remotely triggered, it is not feasible to implement them on wireless medical robots.

**Figure 1:**
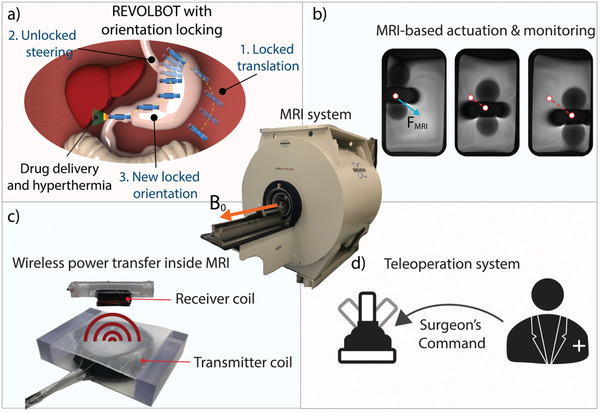
Magnetic resonance imaging (MRI) devices can be used to navigate, steer and monitor wireless magnetic robots remotely in interventional operations while providing high‐resolution soft tissue images. a) Using wireless power transfer‐based heating and a locking/unlocking mechanism, the proposed MRI‐powered cm‐sized capsule robot (REVOLBOT) can translate in 3D in a changeable planar orientation to navigate and steer in tight, narrow, and constrained cavities inside the body. b) An MRI device can provide imaging and actuation. c,d) Under the direct control of a surgeon using a teleoperation control system, the robot has on‐demand hyperthermia and drug release‐based therapeutic functionalities using the wireless heating method and a metal heating tip at the end, which can be used for ulcer treatment in the stomach or tumor treatment in peritoneal cavities in the future. The robot's heating tip and its operation environment can be monitored by fast magnetic resonance (MR) images for safe and precise operation.

REVOLBOT is designed to be near neutrally buoyant, which allows MRI gradients to steer the position of the robot not only in planar tissue surfaces but also in 3D fluid‐filled cavities inside the body. Next, the robot has two orientation‐dependent medical functions, local and on‐demand hyperthermia and drug delivery, towards future clinical applications, such as tumor treatment in peritoneal cavities as depicted in **Figure** **1**. Such functions are enabled by a metallic tip on the robot's one end, which is heated by wireless power transfer using a transmitting coil inside an MRI scanner and a receiving coil on the robot.

In our studies, we monitor the robot using MR images during its teleoperated actuation and control. Typical magnetic robots create MR image distortions that are much larger (e.g., around 10–20 times) than their sizes.^[^ ^31^ ^]^ This image distortion prevents the MR monitoring near the magnetic robots. The presented capsule design that keeps the ferrous magnet distant from the functional end does not prevent monitoring of the target region while the robot is near this region of interest. Preserving such a monitoring capability is essential for the use of miniature magnetic robots in future clinical applications in MRI systems.

## 2 Results

### 2.1 Robot Design

**Figure 2:**
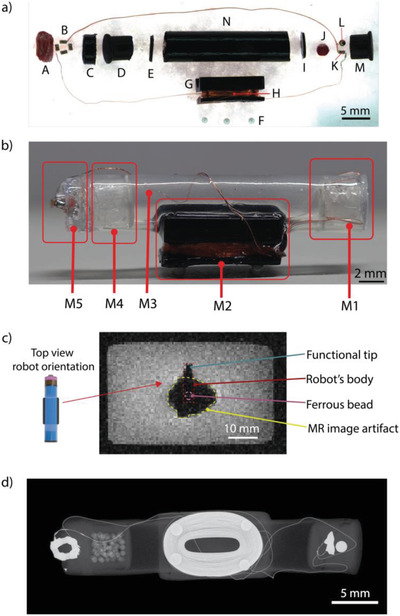
The components, modules, and images of the REVOLBOT prototype. a) Optical microscope side‐view image of all robot components before their assembly. The individual components are: A: meltable drug wax, B: heating unit, C: drug container, D: mass‐balance holder, E: holder cap, F: density‐tuning zirconia ceramic beads, G: inductor frame, H: inductor, I: actuation compartment cap, J: locking/unlocking wax, K: heating unit, L: ferrous spherical bead, M: actuation module holder, N: main body. b) Side‐view photo of the assembled robot with five modules: The module M1 is the actuation compartment consisting of I, J, K, L, and M components; M2 is the inductor coil module consisting of G, H, and F, and used as the receiver for the remote power transfer; M3 is the air gap inside the hollow main body, which is used for density/buoyancy tuning; M4 is the mass‐balance compartment consisting of components D and E; M5 is the functional metal tip end of the robot that consists of A, B, and C for drug delivery and hyperthermia. c) MR image of the robot using the multiple gradient echo (MGE) sequence, where the distorted image is due to the ferrous bead and the functional metal tip is still visible at the left end of the robot image. d) X‐ray image of the assembled robot from the top.

REVOLBOT is a miniature capsule robot manufactured by assembling sixteen distinct parts to integrate magnetic actuation, reversible locking mechanism, drug delivery, and hyperthermia functionalities as shown in **Figure** **2a**. It consists of five main modules: an actuation module, a mass‐balance module, a drug delivery and hyperthermia module, a wireless power receiver coil resonator, and a main body (Figure 2b). The actuation module consists of a 1 mm‐diameter spherical ferrous chrome‐steel bead embedded in heneicosane wax, as a phase‐changing material, and a heating element. The ferrous bead is located close to the tip of the robot to provide rotation to the robot as well as translation with the same MRI magnetic gradients‐based pulling forces in 3D. The heating element and the heneicosane wax region are located in this actuation module to implement a reversible remote heating‐based orientation‐locking mechanism, which leads to a change in the robot's state and kinematics. Further details on the state switching and system dynamics are provided in the next sections.

The robot's center of mass (COM) position is designed to be at the center along its longitudinal axis and shifted downwards along the radial axis towards the gravity direction. Such a shift in the COM position with respect to the center of volume (COV) determines the neutral pitch orientation of the robot inside the 3D fluidic environments. Therefore, the robot always remains horizontally flat due to this intentional COM position shift by the robot design. This design selection will be elaborated more in the robot motion dynamics section.

The mass‐balance module is designed for fine‐tuning of the COM position of the robot along its longitudinal axis. This mass tuning helps the robot stay horizontally balanced inside the fluids without any pitch offset angle. The mass tuning is realized by the inclusion of glass microspheres inside the mass‐balance module. Next, the drug delivery and hyperthermia modules consist of a drug container, a liquid drug (a proof‐of‐concept food colorant in this study for easier visualization), a heneicosane wax region, and a metallic heating tip. This heating element is partially embedded in the wax and partially exposed to the fluidic medium. The heating element is responsible for local hyperthermia and drug delivery by melting the surrounding wax to facilitate the release of the contained drug. The portion of the heating element that is exposed to the fluidic medium is the tip of the robot used for hyperthermia on the contacted target tissue, while the rest of the heating element embedded inside the wax mainly causes the on‐demand drug release.

The wireless power receiver coil resonator consists of an inductor and capacitor tuned to resonate at ≈225 kHz. The lossy elements are the heating points of the resonator. These lossy elements are located at the actuation module and the hyperthermia/drug delivery module. Lastly, the main body is a cylindrical tubing, which connects all of these components together. REVOLBOT is designed to be neutrally buoyant in biologically relevant fluids, e.g., phosphate‐buffered saline (PBS). Due to the hollow shape of the main body, it contains an air gap inside. Therefore, the length of the body is tuned to match the density of the robot at around the PBS density (1.00–1.01 g cm^−3^). Zirconia ceramic beads located under the robot are used for finer density tuning after the robot is assembled.

The 1 mm‐diameter ferrous bead, required for magnetic actuation, creates an approximate 2‐cm diameter MR image distortion around the bead as shown in Figure 2c. This distortion is in the form of signal loss, image warping, and voxel shifts. Therefore, it is not feasible to monitor nearby surrounding regions for a typical robot that consists of only a magnetic bead. This image distortion defeats the tissue and operation monitoring purposes of using MRI systems when the ferrous bead is around the target region. To avoid such a monitoring issue, REVOLBOT's ferrous bead and the functional tip are separated by 2.4 cm distance. This design provides at least a 4‐mm distortion‐free visible region around the functional end of the robot (Figure 2c) for image‐guided safe and precise teleoperation.

### 2.2 Reversibly Switchable Orientation Locking/Unlocking Mechanism

**Figure 3:**
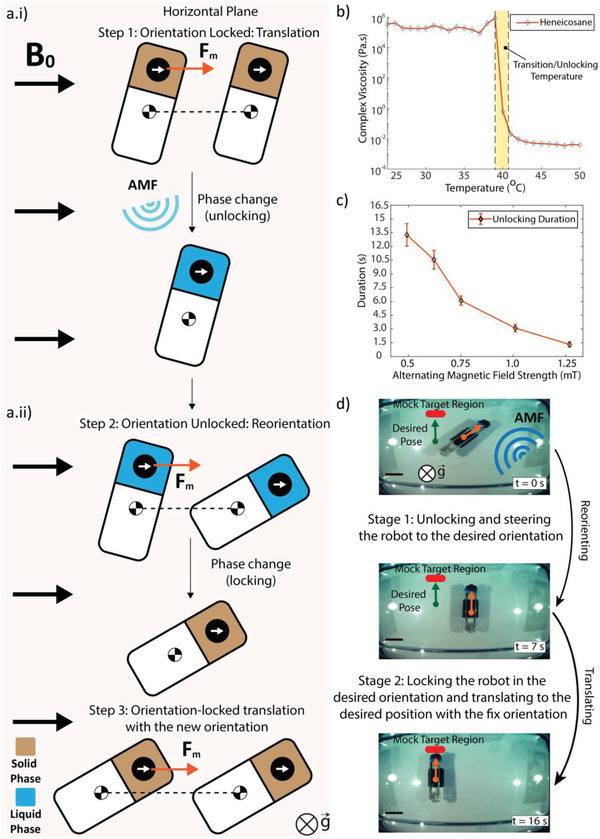
Planar orientation‐change using the locking and unlocking mechanism enabled by the solid‐liquid phase change of the heneicosane wax, in which the ferrous bead is embedded. ai) In the locked state, the wax is in the solid phase, where the ferrous bead and thus the robot body is always aligned with the uniform and very high *B*_0_ field direction. In this state, the robot is translated in 3D in the given locked/fixed planar orientation using the magnetic pulling forces generated by the MR gradients. aii) In the unlocked state, the wax is melted using wireless powering‐based heating to free the ferrous bead rotation, and the applied magnetic pulling forces can now rotate the robot body to a desired planar orientation due to the moment arm with the robot center of mass. After relocking the robot at this desired orientation, the robot can be translated while preserving its orientation. b) Steady‐state viscosity change of the heneicosane wax as a function of the increased temperature, measured by a rheometer. The data shows that the wax melts at around 41 °C. c) Steady‐state unlocking duration for the given robot prototype as a function of the alternating magnetic field (AMF) amplitude, i.e., power. d) Top‐view camera video snapshots taken from Video S1 in the Supporting Information to show the unlocking, re‐orientation, locking, and translation stages of the robot.

REVOLBOT is designed to switch its orientation on demand via remote wireless power transfer‐based heating. With this mechanism, we are able to lock and unlock the robot's orientation by benefitting from the presence of a very strong *B*_0_ uniform field inside the MRI bore. Such a mechanism is realized by embedding the ferrous bead inside a temperature‐based phase‐changing wax (heneicosane) contained in the enclosed actuation module as presented in **Figure** **3a**. Depending on the solid or liquid phase of heneicosane, the robot alignment and the magnet alignment are either locked or unlocked.

By means of permanently present *B*_0_ field, the alignment of the ferrous bead is always fixed along *B*_0_ direction, i.e., along the MRI bore. In State 1 of REVOLBOT, heneicosane is in its solid form. Therefore, the ferrous bead is rigidly attached to the robot body, and the bead maintains the robot orientation at all times. This will be referred as the robot's “locked” state. In State 2, heneicosane is in its liquid form, and the robot can change its orientation, which is referred as its “unlocked” state. The bead is no longer rigidly attached to the robot body and can freely rotate inside the body; therefore, the robot orientation can change under any external torque. Such controlled external torque is induced by magnetic pulling forces created on the ferrous bead by the MRI gradients. Since the bead is located off‐centered, close to the tip, the pulling forces exert a moment at the robot COM (Figure 3a).

Heneicosane is chosen as the temperature‐based phase‐changing material due to its phase transition temperature and its mechanical properties as in liquid and solid phases. Heneicosane goes into a phase transition (melting) at temperatures slightly above body temperature at around 41 °C. Such a phase transition results in a radical change in the material's complex viscosity as presented in Figure 3b. The viscosity change of heneicosane as a function of the applied temperature was characterized in a rheometer (HR‐3, Discovery).

In State 1, the robot orientation is fixed. Any 3D magnetic pulling force vector can cause only 3D translational motion without any rotation on the robot. This state is advantageous if the robot is in the desired orientation and positional steering with a fixed orientation towards a target location is needed. However, in this state, the robot orientation cannot be altered. In State 2, the magnetic pulling vector can cause both position and planar orientation change (yaw rotation). This state should be active if the orientation of the asymmetric capsule robot is needed to be different, or steering with orientation change is needed.

We further investigate the duration it takes to unlock a State 1 (locked) robot. These experiments are conducted in a PBS solution with various alternating magnetic field (AMF) magnitudes, applied by the custom transmitter coil inside the MRI bore, for controlled wireless heating. The PBS temperature for the experiments is kept between 36.4 and 36.8 °C. Since heneicosane melts at around 41 °C, an additional 4–5 °C temperature rise enables the phase change via wireless heating. For the experiments, we sweep the AMF magnitudes in the range of 0.5–1.2 mT root‐mean‐square (RMS). The most rapid state switching occurs at the highest magnitude while the slowest occur at the lowest. Any magnitude below 0.5 mT RMS is not strong enough to induce a phase change of heneicosane and switch the state. The error bars represent the standard deviation for each experiment repeated 5 times. According to the experimental results, the state switching can occur in 1.4 s as the fastest transition and it can be as long as 13.3 s for low‐intensity AMF magnitudes. The results of this experiment are presented in Figure 3c. This experiment demonstrates the range of AMF magnitudes and the required heating durations for the selected five different AMF magnitudes. Exploiting the use of resonators is a common practice for efficient remote heating systems.^[^ ^38^ ^]^ While 1.4 s is the average unlocking duration with the highest AMF power, the average cooling and relocking duration is observed to be 6.21 s.

Sequential and repetitive locking and unlocking of REVOLBOT at various angles ranging from 0° to 180° with 30° increments has been experimented with camera images. The goal is to lock the robot orientations on these desired planar orientations with optical camera images. The robot is controlled by joystick force inputs. The locking orientation accuracy of this experiment is found to be 3.15°. The overall experiment takes 126 s for locking at 6 different orientations as presented in Video S1 in the Supporting Information.

Even though the robot is in a locked state, the yaw orientation of the robot may slightly change depending on the direction of pulling and magnitude. In order to determine the range of orientation variation during the translation of a locked robot, the maximum force strength perpendicular to the robot's longitudinal axis is applied, which maximizes the inserted torque on the robot body. Repeating this experiment for 7 times at different locked orientations (from 0° to 180° with 30° of increments) has shown an average of 3.28° orientation shift with 0.70° standard deviation may occur. The maximum (worst‐case) orientation shift is observed as 4.40°.

State 2 is identical to the kinematics of the robot presented by Erin et al. without any locking mechanism.^[^ ^33^ ^]^ In this state, both position and planar orientation of the robot change is simultaneous, which is not ideal and intuitive in terms of controlling the robot with only force inputs. To eliminate such complexity, we switch the robot into State 1 to maintain its current orientation. Combining these two states allows us to acquire any 3D position and planar alignment with a predetermined pitch angle. With on‐demand switching, the robot can be first steered to acquire the desired final orientation while it is in State 2, then, it can be switched into State 1 to maintain this desired orientation and navigated towards the target position. This actuation sequence is illustrated in Figure 3d and provided in Video S1 in the Supporting Information. The overall actuation sequence starts with a robot locked with a 218.1° orientation. The desired final locked orientation is 270° with a desired 10 mm horizontal displacement. This actuation sequence results in a net COM displacement of 10.5 mm with the final locked orientation of 270.48°. This sequence is completed in 19.6 s with 0.46 mm displacement error and 0.48° locked orientation error. This locking strategy can also help the robot navigate inside the body curvatures and have sharp maneuvers.

### 2.3 Robot Motion Dynamics in Locked and Unlocked States

A ferrous spherical magnet under the magnetic field, *B*, and its gradients, ∇*B*, can experience both force (***F***_m_) and torque (***T***_m_) as

(1) $$T_{m}=V_{m}\;\left(M_{m}\times B\right)$$

(2) $$F_{m}=V_{m}\;\left(M_{m}\cdot \;\nabla \right)B$$

where ***V***_m_ is the magnet volume, **M**_m_ is the average magnetization of the magnet, and B is the magnetic field vector. Euler's laws of rigid‐body motion applied at the COM of the REVOLBOT results in

(3) $$F_{\mathrm{net}}=\;ma\;=F_{m}+F_{d}$$

(4) $$T_{\mathrm{net}}=Ia+\omega \times I\omega \;=r_{m}\;\times \;F_{m}+T_{d}+T_{m}$$

where, in an inertial frame of reference, a is the acceleration of the COM, ***a*** is the angular acceleration of the robot, and **ω** is the angular velocity of the robot. **r**_m_ is the position vector from the COM of the robot to the COM of the magnet. **F**_d_ and **T**_d_ are the disturbance forces and torques, respectively, which could be due to buoyancy, gravitation, drag, and other physical effects. The total mass of the robot is represented by *m*, and the effective inertia tensor is **I**.

Under any slight magnetic misalignment under the 7 T MRI system, **T**_m_ term becomes significant and scales with the misalignment angle. The strength of **T**_m_ can be orders‐of‐magnitude larger than the rest of the other torques presented in Eq. 4 if the misalignment is just higher than 1°. Therefore, Eqs. 1 and 4 indicate that, under the presence of **T**_m_, the robot almost completely preserves its orientation under the static **B**_0_ even though there are other torque components that are orders of magnitude smaller than T_m_ under slight orientation changes.

It is important to note that **T**_m_ is the magnetic torque acting only on the ferrous bead. The reversible state switching mechanism determines the transfer of **T**_m_ from the ferrous bead's body to the robot's body. For a locked robot, **T**_m_ acts on the robot body since the torque on the ferrous component can be transferred into the overall robot body. In such a case, **T**_m_ acts as a resistive dominant torque, it keeps the robot alignment while creating no net torque for the robot to rotate (**T**_net_ =  0).

However, for an unlocked robot, **T**_m_ is not present on the robot's body since the ferrous component cannot transfer the torque on its body to the robot. For locked and unlocked robot states, Equation (4) can be written as

(5) $$T_{\mathrm{net}}=Ia+\omega \times I\omega =r_{m}\;\times \;F_{m}+T_{d}\;\text{(unlocked}\;\text{robot:}T_{m}=\;0$$

(6) $$\begin{array}{ccc} T_{\mathrm{net}} & = & Ia+\omega \times I\omega =\;0\;\text{(locked}\;\text{robot:}T_{m}=V_{m}\;\left(M_{m}\times B\right)\\ & \gg & -\left(r_{m}\times \;F_{m}+T_{d}\right) \end{array}$$

The change in the robot kinematics in different states can also be seen in Equations. (5) and (6). For a locked robot, Equation (6) shows that there is no net torque. This indicates the robot cannot rotate under such a strong magnetic field. REVOLBOT preserves its orientation but it can translate with the magnetic pulling forces generated by the MRI device. On the other hand, Equation (5) represents a net torque depending on the magnetic pulling force exerted via an MRI device. Such a net torque allows the robot to change its orientation by using these magnetic pulling for steering.

**Figure 4:**
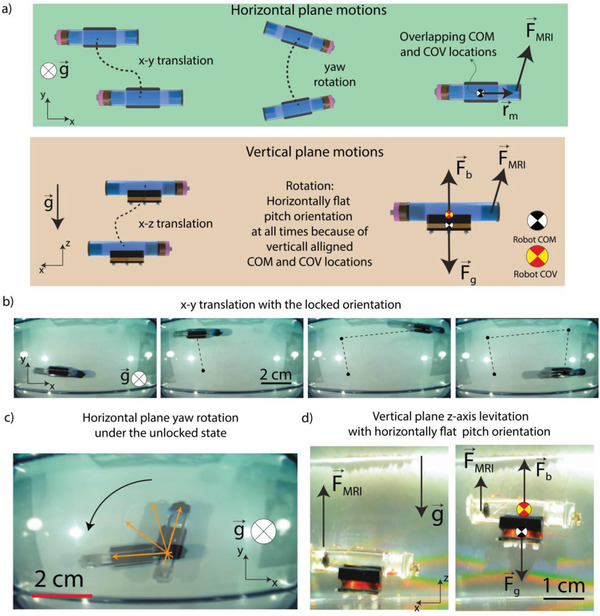
Motion degrees‐of‐freedom of the REVOLBOT. a) On the horizontal plane, REVOLBOT can translate along the *x‐* and *y‐*axis. REVOLBOT under an unlocked state also has the freedom to rotate and change its yaw orientation actively with the magnetic pulling forces generated by an MRI device. On the vertical plane, the robot can be levitated and moved along the*x‐* and *z‐*axis, where *‐z‐*axis is the gravity direction. b) Experimental video snapshots for x and y translation with a fixed orientation. c) Yaw rotation of a robot under the unlocked state. d) Experiment snapshots from Video S1 in the Supporting Information. During the levitation, the robot always stays horizontally flat in the water because of the vertical shift on the position of the robot's COM location (Text S1 in the Supporting Information).

Three decoupled magnetic forces generated by the MRI gradient coils are used to control the 3D position and the planar orientation of the robot. Due to the large vertical shift on the robot COM position compared to its center of the volume (COV) position, the neutral pitch angle of the robot always remains parallel to the horizontal plane even though the robot has the freedom to rotate its pitch orientation. Therefore, the robot can have 4 degrees‐of‐freedom (4‐DOF) active motion control with a pre‐determined constant pitch angle: *x‐* and *y‐*axis translations, rotation in the horizontal plane, and *z*‐axis levitation in the vertical plane (**Figure** **4**). The pitch orientation is fixed as horizontally flat. Therefore, the target application or target region should be reachable while the robot is preserving its horizontally flat pitch orientation at all times.

It is important to note that even though the robot is in the locked state, **T**_m_ does not lock the rotations along the direction of **B**, which can also be interpreted from Equation (1). This is a common case for any magnetic object manipulation due to the fundamental torque equation (Equation (1)). This indicates that a robot at its locked state may still demonstrate a rotational behavior if it is levitated in 3‐D fluids in vertical planes. This unlocked rotational motion in the vertical plane is undesired since it results in indeterministic orientational freedom. In this case, the applied magnetic pulling force may rotate the robot simultaneously while levitating the robot, or an extreme disturbance from the body may also cause a similar undesired rotation. Nonlinear control techniques to approach this underactuated problem may not be feasible due to the limited feedback rate under MRI.

Our proposed robot is designed to have an intentional displacement between its COM and COV to keep this unlocked component of the rotatable orientation deterministically known Text S1, Supporting Information). This shift is designed to be along the gravity direction for the robot located in the horizontal plane. Defining **r**_b_ as the position vector from COM of the robot to the COV of the robot, and **F**_b_ as the buoyancy force, **r**_b_ ×  **F**_b_ term becomes significant when the robot deviates from being horizontally flat in the vertical plane. **T**_d_ includes this buoyancy‐based tilting torque, which is considered as a type of disturbance torque in Equations (4) and (5). Therefore, even though the robot is being pulled against the gravity direction for levitation, the induced torque as **r**_m_ ×  **F**_m_ will be balanced by the torque induced passively by the buoyancy torque and maintain the robot pitch orientation horizontal. Therefore, the robot maintains the designed horizontally flat configuration that is deterministic and known at all times. Orders of magnitude stronger forces could potentially cause pitch orientation deflections. However, for MRI‐powered robots, such a force is not the case. Therefore, the robot maintains its horizontally flat pitch orientation. The levitation of the robot in the vertical plane is also shown in Video S1 in the Supporting Information.

### 2.4 Wireless Power Transfer for Heating‐Based Medical Functions

**Figure 5:**
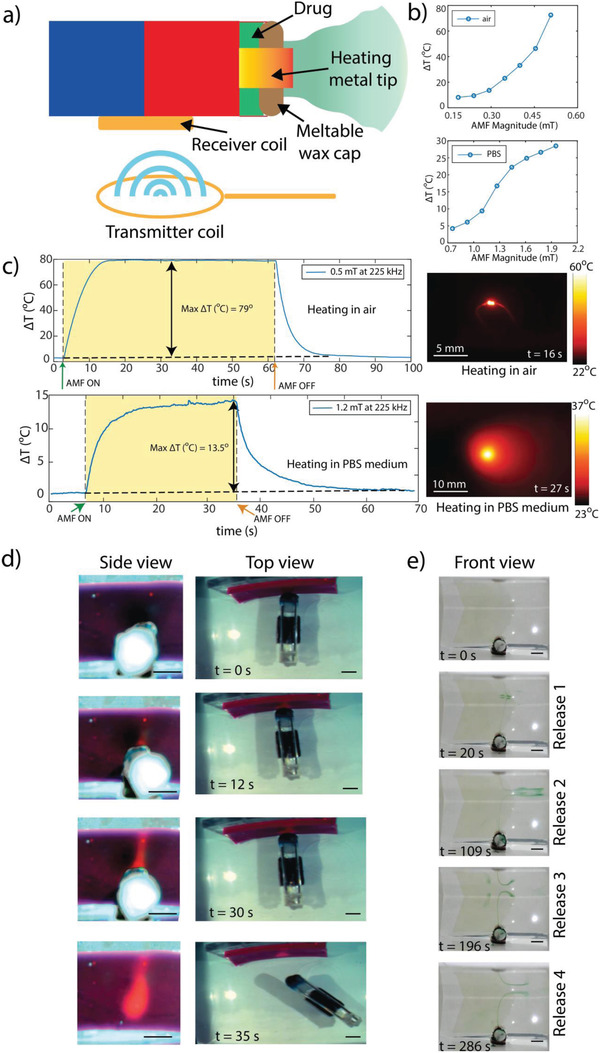
Remote heating‐based on‐demand hyperthermia and drug delivery function demonstrations of the REVOLBOT prototype using its heating metal tip in one end. a) The schematic of the robot showing all robot components and external AMF‐based heating coil, related to the robot medical functions. b) The metal tip temperature increase (ΔT) characterizations in air and PBS solution as a function of the AMF amplitude. c) Heating and cooling dynamics of the heating tip in air and PBS solution when AMF is on and off with 0.5 and 1.2 mT amplitudes, respectively, at 225 kHz. Side images show the infrared (IR) optical microscope images of the robot's heating tip both in air and PBS media (Video S2, Supporting Information). d) Side‐ and top‐view video snapshots of the hyperthermia demonstration of the heating tip in contact with a solid surface. e) Front‐view video snapshots of the on‐demand drug delivery demonstration of the robot, where the melting wax releases the drug molecules (green food dye here for easier visualization) to the fluidic medium by diffusing them from the drug chamber. Such on‐demand drug release is shown 4 times, showing its repeatability and reproducibility (Video S3, Supporting Information).

REVOLBOT is integrated with an LC resonator unit. This LC resonator unit is capable of harvesting resonance‐based wireless power transfer at around 225 kHz and converts this power into heat. The receiver resonator consists of a manually wound rectangular inductor with the dimensions of 10 × 4 × 2 mm^3^ and two separate capacitors (a single capacitor's dimensions are 1 × 0.5 × 0.5 mm^3^) connected in series. The inductive element is fine‐tuned to 1 mH while the overall capacitive element is 500 nF. This wireless heating is used for three main purposes: state switching, hyperthermia, and drug delivery. The harvested power is distributed among two heating elements, which are mostly on the capacitive components with high loss, located inside the actuation module and drug delivery/hyperthermia module. The heating element located in the actuation module is used to control the state of the robot by melting the heneicosane at 41 °C, which results in switching the robot state from the locked state to the unlocked state. The second heating element located in the hyperthermia/drug delivery module is responsible to apply hyperthermia around 43 °C.^[^ ^39^, ^40^ ^]^ Since the drug delivery module has a wax cap melting at around 41 °C, this heating element can also melt this cap and result in drug release on‐demand as depicted in **Figure** **5a**.

The receiver coil is much heavier compared to the rest of the components of the robot. Therefore, the robot COM is vertically shifted towards the inductor, which allows the inductor to remain always at the bottom of the robot and to keep the robot flat while it was floating in 3D inside fluids. Due to this flat orientation, the transmitter coil is located in the horizontal plane to provide the maximum wireless power transfer by keeping the receiver and transmitter coil as parallel as possible. The relative positions of the transmitter and receiver coils as well as the integrated receiver coil on the robot are demonstrated in Figure 5a.

During the experiments and demonstrations, the distance between the transmitter and receiver coil is around 8 mm. The magnetic field used at this distance is 1.2 mT RMS with the resonance frequency of around 225 kHz. This power is used to perform state switching and medical functionalities in PBS at body temperatures. It is important to note that since the hyperthermia/drug delivery tip is exposed to the fluidic medium, the generated heat is dissipated to the environment much faster compared to the heat generated inside the actuation module, which is isolated from the fluidic medium with the plastic robot body. Due to this thermal insulation difference between the wax locations, the unlocking of the robot occurs first. Hyperthermia and drug delivery are observed a few seconds after the robot is unlocked. It should be noted that the extended duration of AMF for drug delivery and hyperthermia keeps the robot in the unlocked state. This is not an issue since the locking and unlocking mechanism is utilized to reach the target destiny with the desired orientation. Once the robot is in the correct pose, longer AMF pulses can be applied to implement its medical functions.

### 2.5 Hyperthermia and Drug Delivery Under MRI

Hyperthermia and drug delivery medical functionalities are selected as proof‐of‐concept demonstrations for REVOLBOT. In clinical settings, it is beneficial to complement chemotherapeutic local drug delivery with hyperthermia for effective tumor treatment procedures, which could increase the uptake of the drug by the tumor region.^[^ ^41^ ^]^ Therefore, we have chosen these two target functions, which are orientation‐dependent since the drug and hyperthermia should occur at the distal end of the capsule robot to be able to monitor them using MR images.

Remote heating characterization experiments are performed in air and PBS medium. The dissipation rate is a key component that determines the maximum temperature for a given transmitted power. Figure 5b demonstrates experimental results for the change of the steady‐state temperature as a function of the pulse magnitude both in air and PBS medium. Similarly, Figure 5c shows the experimental results for the dynamic temperature rise in both air and PBS medium. These results show that 0.5 mT RMS AMF magnitude is sufficient to locally increase the temperature by 79 °C in the air. In the PBS medium, the same resonator realizes 13.5 °C local temperature rise under 1.2 mT RMS AMF magnitude. Since local hyperthermia requires only a 5–6 °C temperature rise, our resonator and the supplied power are strong enough for hyperthermia.

The hyperthermia performance of the robot is demonstrated via temperature‐sensitive color‐changing polymer sheets (Figure 5d). This polymer is capable of switching its color from dark pink to light pink for temperatures above 27 °C. The demonstration experiments are conducted in 26 °C PBS. The robot could lock its orientation to the desired target orientation and then be moved by the MRI gradients with this locked orientation to establish physical contact between the hyperthermia tip and the target polymer region (Video S2, Supporting Information). After wireless powering of the robot for more than 5 s, the effect of heating could be visualized by the color change of the polymer. The heated fluidic medium traveled upward due to convection, which left a trace of color change towards the upper regions on the polymer as well. After applying 30‐seconds‐long hyperthermia under 1.2 mT RMS, the robot is quickly removed from the contact area. The trace of the heated region is observed to be slightly larger than 4 mm‐diameter. The polymer cools down approximately 15 s after the local heating operation. The snapshots from the demonstration are presented in Figure 5d.

Drug delivery experiments are visualized by a green food colorant stored in the drug delivery module of the robot. By heating the tip of the robot, the drug delivery starts occurring within 5–15 s after the AMF was applied. The dye is released to the PBS medium from the first micro‐opening that occurred within the wax cap that started to melt by the increased temperature. The dye release could be realized repetitively as shown in Video S3 in the Supporting Information. In this proof‐of‐concept experiment, the release is triggered 4 times to show its repeatability. Towards the end of the release, the amount of the released dye is reduced. The snapshots of this demonstration are presented in Figure 5e.

### 2.6 Locking Mechanism and Demonstrations Under MRI

One of the most appealing advantages of operating such robots by MRI scanners is to be able to monitor them and the operation environment via MR imaging. Not only tracking the robot but also monitoring its 4‐DOF state and its interactions with the physiological environment is essential for safe and precise usage of these robots in the clinics. Therefore, we developed an MRI sequence that can acquire a single 2D image and then apply a 500 ms of magnetic pulling force consecutively. In order to keep the loop rate high, we used an ultra‐short 2D RARE imaging sequence repetition time (TR) 250 ms long. This yields the overall image refresh duration to be 750 ms. The MR imaging method is selected such that provides the least amount of distortion while providing a high contrast of the mockup experimental setup. Since there is a major trade‐off between the image resolution and the imaging speed, we select ad‐hoc imaging parameters (see Methods and Materials) that compromise between the imaging duration and the resolution.

**Figure 6:**
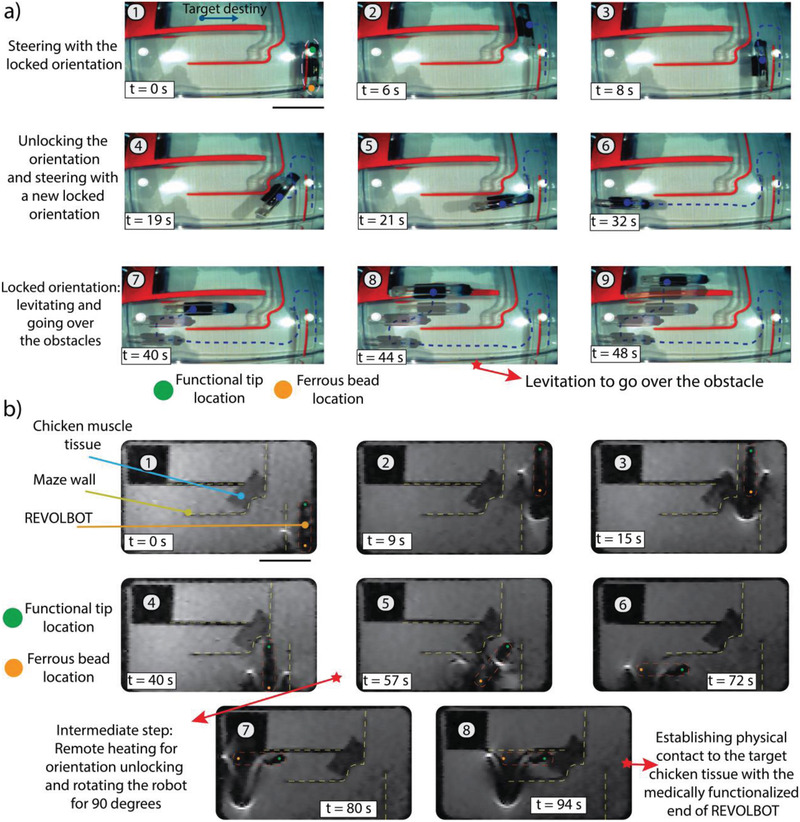
Navigation and steering demonstration of the REVOLBOT prototype using the proposed locking/unlocking mechanism in a synthetic maze while monitoring it by a visual camera and MR scan images. a) Top‐view visual camera image snapshots of the robot: translating up and down in a locked state,^[^ ^1^, ^2^, ^3^ ^]^ unlocking and changing its planar orientation and translating in the new locked orientation to left and up directions,^[^ ^4^, ^5^, ^6^ ^]^ and overlaid video snapshot images going over an obstacle in 3D by levitating the robot using the out‐of‐plane magnetic pulling forces.^[^ ^7^, ^8^, ^9^ ^]^ b) Top‐view MR scan image snapshots of the robot: translating up and down in a locked state,^[^ ^1^, ^2^, ^3^, ^4^ ^]^ unlocking and changing its planar orientation and translating in the new locked orientation to left and up directions,^[^ ^5^, ^6^, ^7^ ^]^ and contacting a chicken tissue in snapshot 8. Here, the robot tip (green colored dot) and the environment can be monitored by MR images during the operation.

In order to provide a ground‐truth demonstration for the robot actuation via MRI gradients, we utilize visual camera feedback for steering it in a synthetic maze embedded in a PBS solution (**Figure** **6**a). The robot can be steered and switched between locking and unlocking states using a joystick‐based control. The robot can be levitated to overcome the obstacles to reach the target area (Video S4, Supporting Information). Such parkour provides a proof‐of‐concept demonstration towards the usage of REVOLBOT in peritoneal cavities, where the space is limited and requires levitation to go over or around the obstacles.

In the second demonstration, we replace the camera feedback with MR image feedback with the sequence mentioned above for teleoperation with MRI. Even though the frame rate is much lower than the camera feedback experiments, the robot can still be steered to the desired location as shown in Figure 6b and in Video S5 (Supporting Information). The elongated robot design allows the functional tip to be visible at any yaw orientation of the robot. In order to demonstrate this visibility, Video S5 in the Supporting Information also presents a teleportative full 360 rotation of a robot with an unlocked state while monitoring under MRI. Since the functional tip is not obscured due to the image distortion of the ferrous bead, clinically relevant operations can be implemented with higher confidence.

The main benefits of the switching between the locked and unlocked states are^[^ ^1^ ^]^ the navigation in confined spaces, and^[^ ^2^ ^]^ being able to approach multiple targets at different orientations. In terms of navigation, by switching the robot's state in a planned manner, the robot can take very sharp turns, which was not possible without this mechanism. In order to demonstrate this navigation capability, we built a maze and navigated robots while monitoring them with cameras under MRI. While REVOLBOT is capable of finishing the designed maze in 31 s, another robot, which does not have such a locking mechanism, experiences more challenges in navigation (Video S6, Supporting Information). This robot cannot proceed further than the first turn since the translation and rotation are always coupled, which results in difficulty in the navigation.

In case of a presence of multi‐tumor scenario, these devices should approach each of these tumor regions individually for treatment. This may require such capsule robots to orient themselves accordingly for each target location and remain in that orientation until reaching the target tumor. In order to demonstrate a scenario for such situations, we placed two moldable‐wax spheres on an artificial stomach model as a mock tumor site. The reversible locking mechanism allows REVOLBOT to approach these multiple target points with different poses in such situations. This demonstration video is provided in Video S7 in the Supporting Information.

## 3 Discussions

The proposed reversible orientation locking mechanism improved the robot's steering and navigation capability in confined and tight regions and enhanced its functionality in orientation‐dependent medical tasks for wireless capsule‐type and other asymmetrically shaped MRI‐powered magnetic robots. Feedback from MR images to monitor the robot position/orientation, interaction with the operation environment and navigation simultaneously are crucial for safe and precise teleoperation.

In this study, we use a preclinical 7T MRI scanner for small‐animal experiments. Compared to the clinical scanners, the magnetic field and its gradients are stronger in our system. Having such a stronger magnetic field has no difference on the actuation of the robot since the chrome‐steel bead used as the actuation component of the robot is saturated in both clinical and preclinical MRI scanners. On the other hand, magnetic gradient pulling forces are about two times stronger since preclinical MR scanners typically have smaller bore sizes. In order to have this study applicable for clinical MRI systems, we limited our gradient strength to 66 mT m^−1^.

Magnetic gradient field strength is an important element that determines the motion capacity of MRI‐powered robots. REVOLBOT is designed to have a 4‐DOF motion in this study. The fifth DOF, which is the pitch orientation, is fixed as horizontally flat by significantly shifting the COM of the robot. With the given gradient strengths of the current system, the robot could perform the fifth DOF if the mismatch between COM and COV locations is less than 46.1 µm. For a cm‐scale robot manually assembled with 16 different components, trying to accomplish this precision may result in indeterministic neutral pitch angles, which affects the remote power transmission and dynamics among various REVOLBOTs. If the magnetic gradient strengths could have been higher, the manufacturing precision requirements would relax and could allow demonstrating a full 5‐DOF motion; however, within the given constraints of the MRI system and REVOLBOT, we designed this study to have 4‐DOF with a fixed horizontally flat pitch orientation.

An implementation of closed‐loop control for REVOLBOT is one of the possible directions for future MRI‐powered robotic studies. Such closed‐loop operation is beneficial if sub‐millimeter precision is needed or the tasks are repeatable and automated. Various systems have been demonstrated for closed‐loop feedback tracking, control, and path planning for magnetic single‐body robots both in stagnant fluids^[^ ^7^, ^11^ ^]^ and under fluid flow.^[^ ^9^, ^32^ ^]^ However, these systems are capable of demonstrating up to 3D position control, therefore, orientation control with such a locking mechanism remains as an open field of research. With this presented locking and unlocking mechanism, an additional degree‐of‐freedom to this underactuated system is introduced to improve the controllability of such MRI‐powered capsules.

The low feedback rate due to slow MR imaging is a challenge for robust and precise autonomous control in especially dynamic environmental interaction and flow conditions for extended degrees‐of‐freedom control. By employing 1D imaging^[^ ^12^ ^]^ or fast MR imaging techniques,^[^ ^42^, ^43^ ^]^ this feedback rate could be increased up to tens of Hz. The magnetic signature selective excitation tracking (MS‐SET) technique is the most promising 1D imaging method for such a high position update rate.^[^ ^44^ ^]^ However, the poor position accuracy of this technique could induce performance reduction for the model‐based control methods and trajectory planning‐based precise applications.^[^ ^12^ ^]^ Therefore, the future of autonomous robotic applications should consider many concepts together, such as MR imaging and actuation requirements, along with the trade‐off between the imaging duration, tracking accuracy, control methodology, and tissue monitoring capabilities.

The current REVOLBOT monitoring, steering, and actuation feasibility demonstrations are conducted in stagnant fluid conditions. However, it is crucial to investigate the closed‐loop control when fluidic conditions are dynamic and navigating in tight 3D environments. For single‐body MRI‐powered magnetic particles, the position control of these particles has been shown to navigate them in 3D vessel phantoms with the pulsatile flow as in blood streams.^[^ ^32^ ^]^ Extending such control techniques for also orientation control would be helpful for autonomous tasks.

Here, we show proof‐of‐concept hyperthermia and drug delivery functions for REVOLBOT using resonance‐based wireless power transfer. With such on‐board power, other medical diagnostic sensors (e.g., camera, biosensors) and therapeutic functions (e.g., tissue or fluidic biopsy)^[^ ^45^, ^46^ ^]^ can be integrated into the robot body as future work. For these functions, one‐way or two‐way wireless communication could be needed using AMF waves or other methods. Moreover, the current methods need to be validated in *in‐vivo* animal experiments in the future, where fluid flows, organ motions, and tissue background noise in MR images need to be compensated by optimizing the presented imaging and control methods, towards future clinical applications.

## 4 Experimental Section

### Design and Manufacturing of REVOLBOT

The miniature capsule‐type design of REVOLBOT is accomplished by assembling sixteen components including both mechanical and electrical components. The drug container, mass‐balance holder, actuation module holder, holder cap, and actuation compartment cap are printed via Connex3 and Objet260 3D printers. The inductor frame is 3D printed by Carbon M2 due to the enhanced rigidity required for winding the coils. The main body is a 2.1‐cm long pellethane tubing (Nordson Medical, Part No 115–1809) extracted by cutting with a sharp knife from a longer tubing. The ferrous bead is a 1‐mm in diameter chrome‐steel bead (Kugel‐Winnie GmbH). The temperature‐based phase‐changing wax used for locking/unlocking and drug delivery mechanism is heneicosane (Sigma‐Aldrich, 286052‐10G), which has a melting temperature at around 41 °C, to be compatible with *in‐vivo* conditions in the future. The resonator consists of capacitive elements (Knowles Syfer, Part No 6 565 299) and an inductive element, which was a 350‐turns coil with a 50 µm‐thick copper wire hand‐wound at around the 3D‐printed inductor frame. This inductive element is also the receiver element for the resonator. In order to distribute the dissipated power for multifunctional purposes, the inductor is connected into one single lossy capacitor and a 2 × 2 lossy capacitor array in series. The single capacitor is inserted in the actuation module to realize the unlocking mechanism, while the 2 × 2 capacitor array is attached to the drug release module and hyperthermia to realize these medical functions.

The assembly process of the robot includes many steps performed under the stereo optical microscope (Stemi 508, Zeiss) for higher precision. First, the hand‐wound inductor coil is soldered onto a single capacitor and a 2 × 2 capacitor array in series. The single capacitor lead is left open at this stage to be connected to the 2 × 2 capacitor array in series. This lead connection is done as the final step of the assembly. Then, the mass balance module and the actuation module are assembled. The mass‐balance module is assembled by partially filling the inside of the mass‐balance holder with glass beads (Sigma‐Aldrich, G8772‐10G) and using cyanoacrylate‐based adhesives to attach the holder cap. For assembly of the actuation module, the ferrous bead and the single capacitor are inserted in the actuation module holder.

With the help of a pipette, a 15 µL volume of melted heneicosane is injected inside of the module. The wax is cooled down until it solidified inside the module. The wax starts to solidify immediately after it contacts the components inside the module, which prevents the wax to spread evenly inside the module. In order to make sure the heneicosane is well‐spread and holds the ferrous bead while the capacitor is embedded in the wax, the actuation module is heated on a hot plate until the wax is melted. The position of the single capacitor and the ferrous bead is also improved in this stage while the heneicosane is in its liquid state. The actuation compartment cap is attached at the top with a cyanoacrylate adhesive (402, Loctite) to complete the assembly of the actuation module.

The inductive component is attached to the surface of the main body by using the cyanoacrylate adhesive. At this stage, previously prepared actuation and mass‐balance modules are also inserted inside the tubing from both ends. In addition to the tight fit assembly of these modules inside the main body tubing, the cyanoacrylate adhesive is applied around the assembly surfaces to ensure a strong attachment. The 2 × 2 capacitor array is attached in the center of the drug holder, and the drug holder module is then attached behind the mass‐balance module with the cyanoacrylate adhesive. The leads left open during the resonator manufacturing is connected at this stage. The minimum lead length needed is the length of the robot's overall body. However, in order to avoid stretching‐related damages in the resonator, the lead wires are left longer and wrapped around the body to accommodate this excess length. The open lead end is soldered onto the already attached 2 × 2 capacitor array. The overall resonator is coated with lacquer (PLASTIK 70, Kontakt Chemie) to ensure the sealing of the assembly and electrical insulation of the resonator. As the final step, the 10 µL mock drug is injected inside the drug holder module with a pipette and the overall drug module is sealed by a heneicosane wax.

The relative location of the COM and COV of the robot determines the neutral pitch angle of the robot immersed in the fluid. Designing a robot with coinciding COM and COV locations is not desired since micron‐level manufacturing uncertainties result in an indeterministic neutral pitch angle of the robot. In order to have a more deterministic pitch angle, REVOLBOT is intentionally designed to have a large radial distance between the COM and COV locations of the robot which results in horizontally flat neutral pitch angles (Text S1, Supporting Information). Such a horizontal pitch angle keeps the receiver and the transmitter coil in parallel and maximizes the power transfer between the coils for effective heating. Additionally, the robot's pitch angle remains deterministic and horizontal during the robot operation. We accomplish such a radial COM shift by attaching the resonator at the bottom of the robot. Since the inductive element of the resonator is the heaviest component of the robot, the robot's COM becomes significantly shifted, and therefore, the robot's neutral angle remains mostly horizontal with the inductive element located at the bottom.

### Magnetic Pulling Forces inside MRI

Due to the constant and strong magnetic field (***B***_0_) of the MRI devices, the ferrous bead is magnetically saturated and it is magnetically aligned always with the *z*‐direction of ***B***_0_. The magnetic field of MRI devices and the bead magnetization vector (**M**_m_) can be represented as

(7) $$B_{0}={\left[\begin{array}{ccc} 0 & 0 & B_{0} \end{array}\right]}^{T}\;$$

(8) $$M_{m}={\left[\begin{array}{ccc} 0 & 0 & m_{m} \end{array}\right]}^{T}\;$$

where *B*_0_ represents the strength of the strong unidirectional magnetic field, and *m_m_* is the magnetization strength of the ferrous bead. Inserting Equations (7) and (8) into Equation (2) reveals the magnetic pulling force under MRI as

(9) $$F_{m}=V_{m}\;m_{m}{\left[\begin{array}{ccc} \frac{\partial B_{x}}{\partial z} & \frac{\partial B_{y}}{\partial z} & \frac{\partial B_{z}}{\partial z} \end{array}\right]}^{T}$$

This pulling force with three orthogonal and independent force components can be generated by MRI gradient coils along any 3D direction and any magnitude within the specifications of the instrument.

### Remote Heating System and Integration Under MRI

The remote heating system consists of a 24‐turn transmitter coil with a 6‐cm diameter. The coil is powered by a broadband amplifier (BBA 150, Rohde & Schwarz), which is fed with a signal generator unit (DSG3060, RIGOL) around 225 kHz sinusoidal shape signal. The resultant alternating magnetic field signal at 8‐mm away from the isocenter of the transmitter coil is up to 1.2 mT RMS at around 225 kHz frequency. This frequency matches with the receiver resonator's frequency to maximize the power transfer and efficiency for remote heating.

Powering the transmitter coil for remote heating does not cause any observed hardware issue on the MRI hardware and its coils. However, while the amplifier is turned on (not necessarily powering the transmitter coil) the MR imaging signal is deteriorated. Therefore, MR images show a very low signal‐to‐noise ratio during the wireless power transfer.

The transmitter coil is placed under the experimental workspace. Since the robot's receiver is at its bottom parallel to the horizontal plane, such a location gives the highest power transfer efficiency. Since the transmitter coil is 6‐cm in diameter, the unlocking and medical functions should be triggered inside of this region in the robot operation space.

The heating characterization experiments in PBS and air medium are monitored via infrared camera (ETS 320, FLIR) to monitor and record the maximum temperature created. These characterization experiments are conducted outside of the MRI. The robot is located at the center of the coil, with the same distance away from the surface of the transmitter coil as it is in the MRI demonstrations. These characterization experiments provide us the feasible range of AMF magnitude needed for hyperthermia.

### Sequential Imaging and Actuation via MRI

The MRI gradient coils generate 3D magnetic gradient fields with a very high spatiotemporal resolution, which is used mostly for spatial encoding of the MRI signals. The same gradient fields are used to actuate ferrous objects by exerting a remote pulling 3D force on them. In this study, imaging and actuation cycles are designed sequentially. Therefore, MRI gradient coils hardware is shared for imaging and actuation purposes sequentially.

We optimized the imaging sequence to provide clear and sufficient monitoring capability while keeping the duration short enough to boost the image refresh rate. The main trade‐off is between the image resolution and the imaging duration. For MRI monitoring, we use Rapid Acquisition with Relaxation Enhancement (RARE) sequence with 128 × 128 image size. The acquired images are 2D with the slice thickness of 2 mm, and the field of view is 10 × 10 cm^2^. TR and effective echo time (TE_eff_) are 250 ms and 40.50 ms, respectively. The echo time is 4.5 ms and the rare factor is 38.

3D actuation forces generated by MRI gradient coils are controlled via joystick inputs. In each imaging/actuation cycle, the magnitude and the direction of the force remain constant. In order to balance between imaging and actuation, we use a 500 ms‐long actuation period. This results in a 750 ms‐long full‐cycle duration. Therefore, the image refresh rate in our experiments is 1.33 Hz.

### MRI Software and Hardware Setup

The software communicated with the MRI hardware is based on ParaVision (Bruker), which has a user interface for developing and using various MR imaging sequences. We develop custom consecutive imaging and actuation pulse programs in this software environment. A custom MATLAB script is used to modify the actuation gradient values on‐the‐fly. Any magnetic gradient force inquiry raised by this script is transferred into the ongoing pulse sequence for the next cycle. The script is capable of providing any desired actuation signals. In our experiments, we integrated a PlayStation console to provide user actuation signals with intuitive joystick commands. This on‐demand and arbitrary open‐loop actuation enable us to accomplish proof‐of‐concept tasks for experiments under various conditions.

The MRI hardware system used in this study is Bruker, BioSpec 70/30 with quadrature birdcage coil 198/154 mm (outer/inner diameter). This preclinical MRI system has a 30‐cm bore with a 30‐cm long magnetically uniform region. A phantom holder is 3D printed and then integrated on a sliding stage to push the phantom holder in‐and‐out. Two orthogonally aligned MRI compatible cameras (MRC, 12M) and two MRI‐compatible LED light units are integrated on a 3D‐printed frame, which is inserted as a tight fit inside the MRI bore. This frame is kept stationary inside MRI and the cameras located on it are utilized for optical recording of the experiments. The sliding stage holds the experimental phantoms and workspaces. For the recording of the experiments under MRI, the sliding stage is being pushed inside of the MRI device to bring the experimental setup under the imaging location (either for optical recording or MR imaging).

## Conflict of Interest

The authors declare no conflict of interest.

## Author Contributions

O.E. and M.S. conceived and proposed the research. O.E. designed the experimental layout, analyzed all data, and conducted the experiments. O.E. and M.S. wrote the manuscript, with some assistance of M.B. O.E. fabricated the capsule robot. M.B. designed and fabricated the wireless power transmission‐based remote heating system. O.E. and M.B. conducted the remote heating experiments and characterizations. O.E. and M.E.T. established the MRI open‐loop control software and pulse sequences. O.E., M.B., and J.L. carried out the MRI experiments. All authors reviewed and edited the manuscript.

## Supporting information

Supporting Information

Supplemental Movie 1

Supplemental Movie 2

Supplemental Movie 3

Supplemental Movie 4

Supplemental Movie 5

Supplemental Movie 6

Supplemental Movie 7

## Data Availability

The data that supports the findings of this study are available in the Supporting Information of this article.
