# Supplementary material for: Wireless MRI‐Powered Reversible Orientation‐Locking Capsule Robot
Source: Adv Sci (Weinh). 2021 May 3;8(13):2100463. doi: 10.1002/advs.202100463 (PMC7612672; doi:10.1002/advs.202100463)
Supplement: Supplementary file 1 — Supporting Information [file ADVS-8-2100463-s004.pdf]

© 2021 Wiley-VCH GmbH

ADVANCED SCIENCE
Open Access

Supporting Information

for *Adv. Sci.*, DOI: 10.1002/advs.202100463

Wireless MRI-Powered Reversible Orientation-Locking Capsule Robot

*Onder Erin[1,2], Mustafa Boyvat[1], Jelena Lazovic[1], M. Efe Tiryaki[1,4], Metin Sitti[1,3,4]**

Supplementary Materials for

Wireless MRI-Powered Reversible Orientation-Locking Capsule Robot

Onder Erin[1,2], Mustafa Boyvat[1], Jelena Lazovic[1], M. Efe Tiryaki[1,4], Metin Sitti[1,3,4*]

[1] Department of Physical Intelligence, Max Planck Institute for Intelligent Systems, Stuttgart, Germany.

[2] Department of Mechanical Engineering, Carnegie Mellon University, Pittsburgh, PA, USA.

[3] School of Medicine and College of Engineering, Koç University, Istanbul, Turkey.

[4] Department of Information Technology and Electrical Engineering, ETH Zurich, Zurich, Switzerland.

* Correspondence to: sitti@is.mpg.de

The PDF file includes:

Text S1: Mass and density information of the materials used for the robot manufacturing.

Text S2: Physical setup inside MRI with cameras.

Text S3: Physical setup of remote AMF heating system.

Text S4: Temperature changing polymer heat map pictures.

Text S5: MRI-based imaging and actuation sequence.

Fig. S1: Mass and density distribution of REVOLBOT.

Fig. S2: Physical setup inside MRI with cameras.

Fig. S3: Physical setup of remote AMF heating system.

Fig. S4: Temperature changing polymer heat map pictures.

Fig. S5: MRI-based imaging and actuation sequence.

Table S1: Mass and density information of the materials used for the robot manufacturing.

Captions for movies S1-S7.

Other Supplementary Materials for this manuscript include the following:

Movies S1-S7 (mp4 format)

Supplementary Text

Text S1. Mass and density information of the materials used for the robot manufacturing

The antiparallel buoyancy force and the gravitational force acting on the robot body determine the force needed to levitate the robot. With gradient magnetic pulling forces, it is possible to accomplish tasks in three-dimensional workspaces, where a levitation force is a must to advance further towards the target goal. Therefore, mass of each component and the overall volume of the robot is important. The measured mass amount is presented in **Table S1.** REVOLBOT is manufactured by assembling 16 different components. Among these components, the length of the main body kept variable to adjust the air gap size inside the robot. After manufacturing, the robot is intentionally kept to be neutrally buoyant. This slight buoyancy is tuned with high density ceramic bead attachments under the robot.

In addition to the overall density of the robot, it is also very important to know the exact location of CoM of the robot relative to the CoV of the robot. The relative positions of these two points determine the natural tilt angle of the robot immersed in the fluid. The natural tilt angle is desired to be horizontal for our designed robot because this orientation aligns transmitter and receiver coil to be parallel to each other. Also, the orientation-dependent medical functions at the tip can be best practiced with such a tilt angle. Moreover, it is desired to have the robot to preserve this tilt angle at all times. In other words, it is desired to keep the horizontal tilt angle while MRI-powering or external disturbances acting upon the robot body.

In order to meet this criterion, REVOLBOT is designed in a way that CoM of the robot is drastically shifted towards the receiver coil module vertically as shown in **Fig. S1**. Additional ceramic beads used for density tuning located under the receiver coil to further improve such CoM shift towards the bottom. Thanks to this design, the robot's tilt orientation is ensured to be very close to the horizontal orientation. Moreover, this tilt orientation is preserved while moving the robot in three-dimensional volumes and it provides robustness to the external disturbances.

Mass measurements for each individual components of the robot

Component Name	Unit Mass (mg)	Quantity	Result (mg)
**Wax Seal Cap**	28.16	1	28.16
**2x2 Capacitor**	15.32	1	15.32
**Drug Container**	20.17	1	20.17
**Mass Balance Holder**	27.3	1	27.3
**Mass Balance Cap**	6.57	1	6.57
**Ceramic Bead**	3.85	10 (tunable)	38.5
**Inductor Frame**	122.37	1	122.37
**Wound Coil**	161	1	161
**Actuation Module Cap**	6.57	1	6.57
**Switching Wax**	1.5	1	1.5
**Single Capacitor**	4.18	1	4.18
**Ferrous Bead**	4.25	1	4.25
**Actuation Module Holder**	27.3	1	27.3
**Main Body**	82.37	1	82.37

**Liquid Drug**	8	1	8
**Glass Bead**	8.5	1	8.5
**Lacquer Coating**	70	1	70
**Total Mass (mg)**			632.06
**Total Volume (mL)**			0.62
**Net Density (kg/m^3)**			1019.45161

Table S1. Mass, volume and density information of the manufactured REVOLBOT prototype and its components. The robot's density is tuned by introducing high-density ceramic beads. The resultant robot density is close to the PBS medium density but slightly heavier.

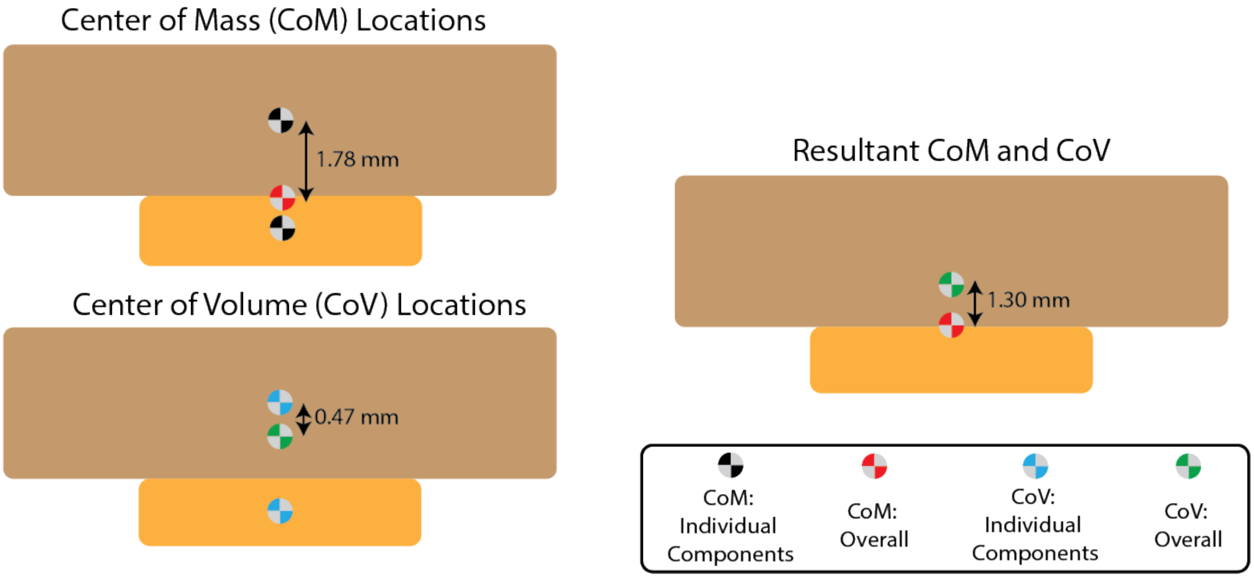

Fig. S1. The proposed robot is able to move in 3D fluids. For a buoyant robot immersed in water, locations of CoM and CoV determine the robot's natural tilt angle in the fluid. Since the desired pitch orientation is horizontal, an intentional and large displacement between CoM and CoV along the robot's short axis is introduced to the robot design. In this figure, the corresponding CoM and CoV for the main body and resonator components are depicted. Also, the overall CoM and CoV volumes with calculated locations with relative distance between them is provided. With such mass distribution, REVOLBOT is capable of staying horizontally flat during the actuation.

Text S2: Physical setup inside MRI with cameras

In order to visualize the robot motion inside MRI, an auxiliary camera system is designed and built inside MRI. This recording system consists of two MRI compatible cameras and two LED lights. These four components are assembled on a 3D-printed frame, which is located inside the bore. The frame is designed to have a tight fit inside the bore to have a fix location. The experimental stage can be pushed inside the scanner until it is located under the workspace of the two cameras. The cameras are placed orthogonally. This provides a top view and a side view for the experimental pools. A video can be captured simultaneously via computers located in the MRI control room. The physical system images are provided in **Fig. S2**.

In addition to the camera system, we also built an MRI-sliding insert unit to ease the experimental preparations. The MRI system comes with a sliding unit to push the samples in at the center of the bore. We designed an alternative experimental stage attached to the slider. The stage is designed to utilize the region of interest of the camera system as much as possible inside the animal scanner. This design also enables us to add additional modules such as transmitter coils, or heating pad for pool temperature adjustments within the limited space inside the bore.

This experimental stage is designed to be 80-cm long to be able to reach the isocenter of the MRI bore. Due to its long length and cantilever beam attachment of the stage, 1-cm thick acrylate is used to provide sufficient rigidity.

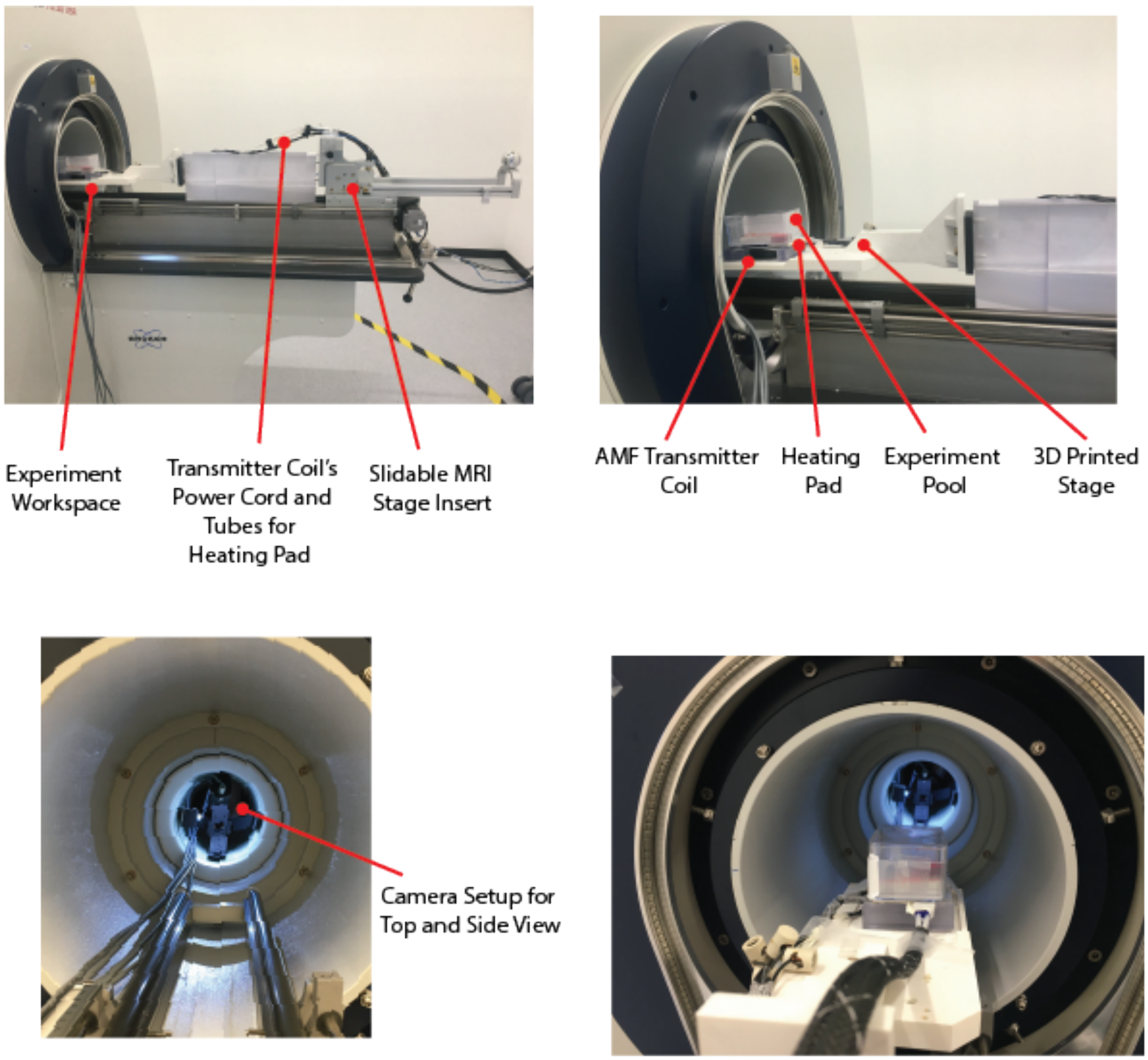

Fig. S2. Photos of the experimental setup for the MRI experiments. The 3D-printed stage is integrated onto the slidable MRI stage insert. This stage embodies the experimental workspace, water tubes and power cords. The experimental workspace consists of the AMF transmitter coil, a heating pad and an experiment pool. A fix camera setup with LED illuminations for top view and side view is located inside the MRI. Prior to the experiments, the stage insert is slided towards inside of the MRI, where the experiment workspace is located in the isocenter of the MRI under the cameras.

Text S3: Physical setup of the remote AMF heating system

The remote powering system consists of (1) transmitter coil, (2) signal generator and (3) broadband power amplifier. The transmitter coil located under the experimental workspace. This coil is connected to the broadband power amplifier. The transmitter coil resonates around 225 kHz inside the MRI bore. This is in the range of our excitation frequency for the miniature capsule's remote heating functionalities. The broadband power amplifier (Rohde & Schwarz, BBA 150) amplifies the signal received by the signal generator (RIGOL, DSG3060) and powers the transmitter coil.

The signal generator provides a low power signal to the broadband power amplifier. We use sinusoidal signal as the input to the broadband power amplifier. The frequency and the peak-to-peak amplitude are the two parameters we are able to control with the signal generator. Since we use the resonance frequency of the transmitter, the amplification power we needed is the minimum. The physical setup for the remote AMF heating system is provided in **Fig. S3**.

The transmitter coil geometry determines the inductance value and the physical region of the remote powering. Due to the considerations of the limited workspace and experimental demonstration region of interests, the diameter of the transmitter coil is designed to be 6 cm. The coil consists of 22 loops with 1 cm thickness. Capacitive components are connected in series with the transmitter coil for resonance frequency tuning and matching. The transmitter coil system is inserted in a 3D-printed coil holder to provide flat attachable surfaces. The coil holder attached into the experimental sliding stage with a double-sided tape. The experimental pool and heating pad are attached on the top of the coil holder with a double-sided tape as well.

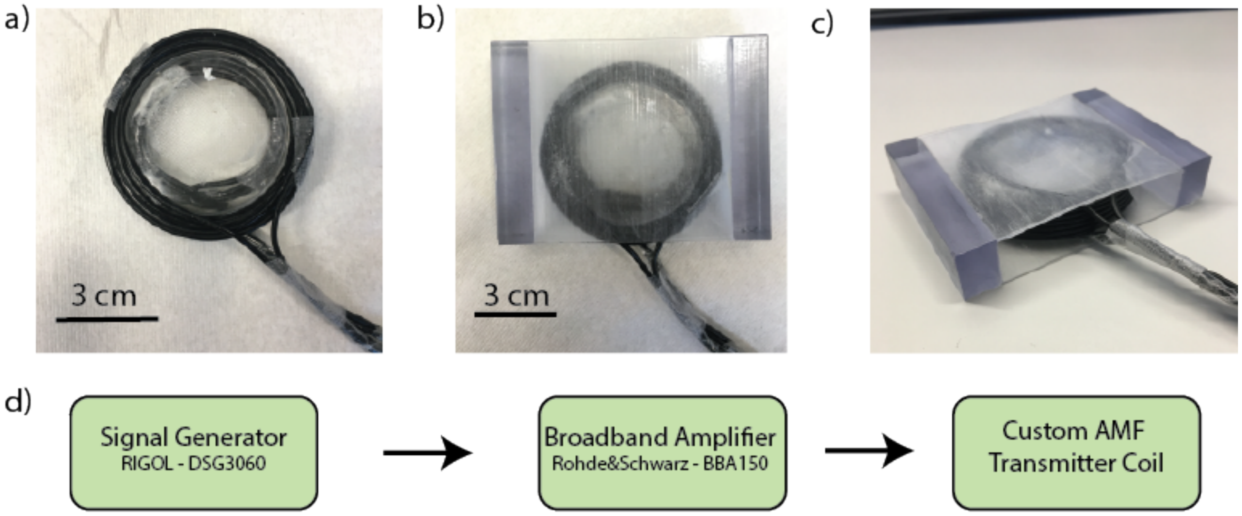

Fig. S3. a-c) Different view photos of the custom-made AMF transmitter coil. This coil in inserted in a 3D-printed coil holder to provide flat surface for experimental pool container. d) Schematic for the power electronics used for the powering of this coil. A signal generator is responsible to dictate the shape of the remote heating signal. The broadband amplifier is responsible to provide sufficient power to the custom transmitter coil by amplifying the signal from the signal generator.

Text S4: Temperature changing polymer heat map pictures

Real-time remote heating demonstrations are visualized by using thermochromic soft surfaces embedded in PBS media. A commercially available thermocromic powder is mixed with Ecoflex 00-30 to create a soft thermochromic surface for experiments. A flat, 3-mm thick polymer surface is cut with a sharp knife and transferred onto a rigid plastic backing, and then attached inside the experimental pool by using cyanoacrylate adhesives. The color of the thermochromic surface is dark pink at cold temperatures. The color changes into light/bright pink under high temperatures. The most drastic color change occurs at around 28 ^{O}C. In order to provide the range of spectrum, we mapped the color to the temperature by using IR camera to measure the temperature and taking visual camera images under the same lighting conditions. These mapping images are provided in **Fig. S4**.

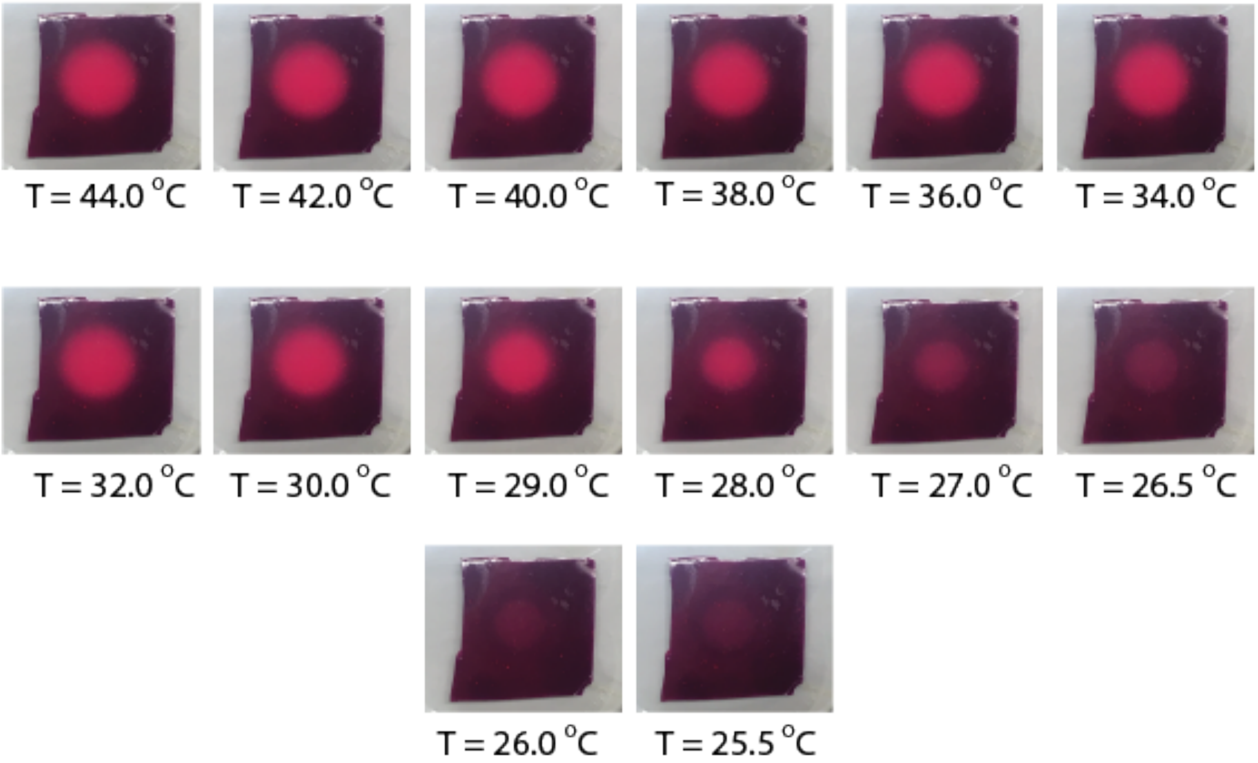

Fig. S4. Thermochromo polymer used in experiments changes its color as a response to temperature. At cold temperatures, the color is purple. At high temperatures, the color is pink. The most observable color change occurs at around 28°C.

Text S5: MRI-based imaging and actuation sequence

In order to provide visual feedback for teleoperation in nontransparent enclosed regions in human body, we developed an imaging sequence capable of providing 2D MR images and remote actuation of the untethered robots. This sequence consists of two phases. (1) 2D imaging phase is a typical imaging sequence used for MR scans. (2) Actuation phase is the phase that a surgeon exerts force on the magnetic capsule for teleoperation via commanding to the MRI hardware. The acquired 2D image is recorded in a text file on the MRI computer. This text file is opened and visualized by a custom MATLAB script swiftly. The operator is able to monitor the robot with a certain image refresh rate. The 2D image update frequency is equal to the loop frequency. The loop duration is equal to $T_i + T_a = T_L$, where T_i is the imaging duration, and T_a is the actuation duration. Therefore, shorter the T_i and T_a, higher image update rate.

However, shortening these subphases comes with a trade-off. Shorter T_a while keeping T_i constant means the net average force exerted on the magnetic bodies is less since the net force F_{net} can be represented as $D \times F_{MRI} = F_{net}$, where D is the duty ratio: $D = T_a/(T_i+T_a)$. Similarly, having a shorter T_i is another approach to increase the update rate on the images. Typically for a selected imaging sequence, T_i is highly dependent on TR (repetition time) and TE (echo time) as well as the image resolution. For any selected imaging sequence, there is an optimum duration for each of these times, which creates the best contrast for the imaging purposes. In our case, we decided to reduce the TR and resolution as much as we can, where the images acquired are still clear enough to monitor the robot state and to steer it towards the target *ex-vivo* tissue. The imaging sequence we used is RARE, the TE and TR parameters are 40 ms and 200 ms, respectively. The resolution of these images is 96x96 pixels. The actuation phase duration is selected to be 400 ms. Therefore, the total cycle duration and the image refresh rate, T_L, is around 600 ms for the demonstrated experiments.

A single computer is being used for both sending teleoperation actuation commands to the MRI system, and also to visualize the status of the robot via MR scans. A MATLAB script, which modifies the actuation parameters and visualizes when there is a new MR scan, runs in a loop. The teleoperation commands are given by a joystick via an operator. Simultaneously, the ParaVision software, which is the main software to communicate with MRI hardware in high temporal and spatial commands, runs our designed sequence in a loop. This designed sequence loop consists of the aforementioned imaging and the actuation cycles. The imaging cycle is preset and we do not need to change its parameters. However, the actuation sequence runs the three actuation parameters that is overwritten on-the-fly by our custom MATLAB script that simultaneously runs in the background. This system's schematic representation for the signal flow and hardware control is depicted in **Fig. S5** below.

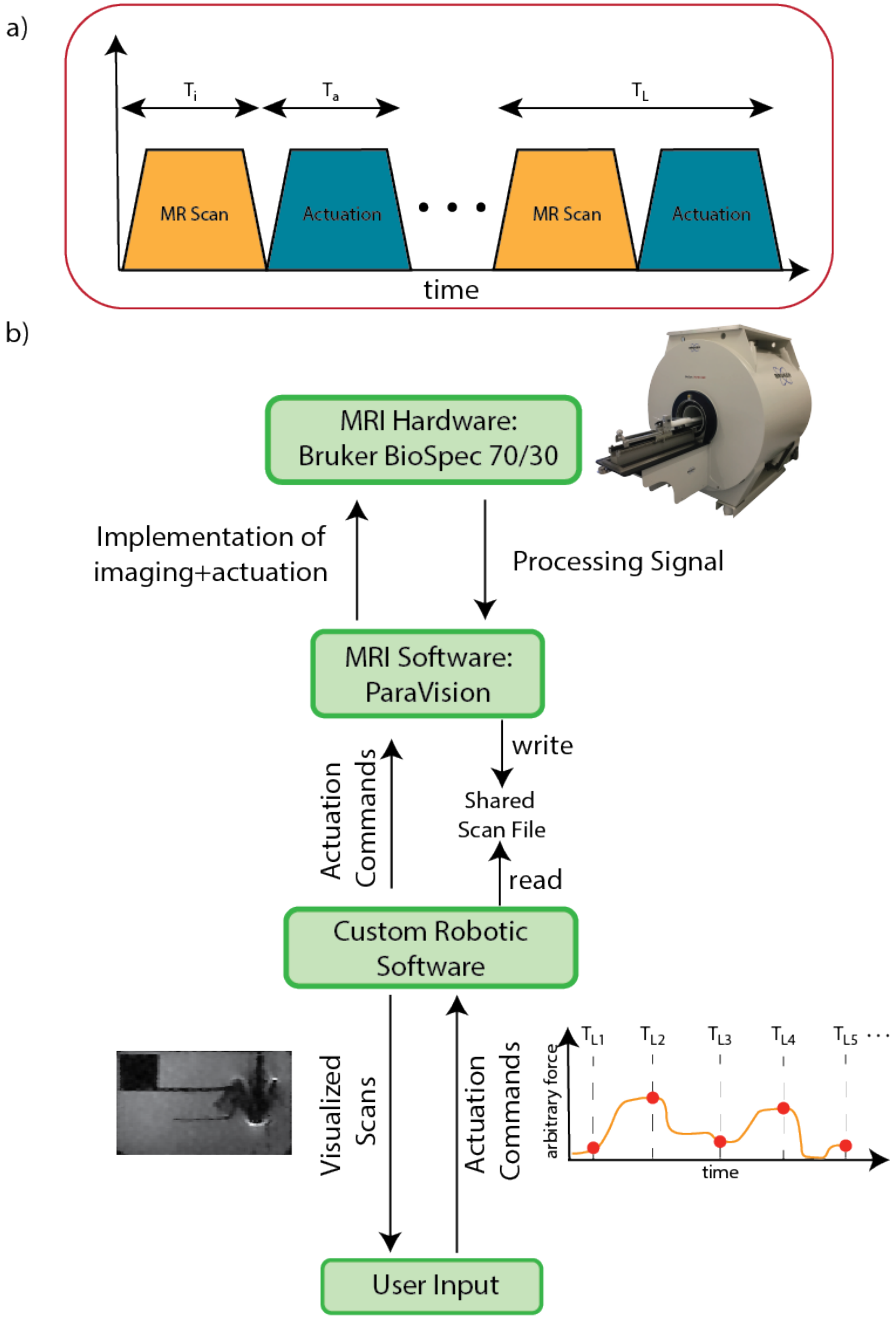

Fig. S5. a) General structure for the sequential imaging and actuation frame with an MRI device. The MRI gradient coil hardware is being used for a dual purpose sequentially over time. This cycle repeats itself until the user stops the sequence. b) Schematic of the high-level system used for MR monitoring and actuation. Major nodes are represented with green boxes.

Supplementary Movies

Movie S1 (.mp4 format). Locking/unlocking mechanism and robot motions.

Movie S2 (.mp4 format). Steering and remote heating demonstration under MRI.

Movie S3 (.mp4 format). Drug release demonstration.

Movie S4 (.mp4 format). MRI-powered steering in a maze.

Movie S5 (.mp4 format). MRI monitoring and MRI steering demonstration.

Movie S6 (.mp4 format). Comparison between robots with locking mechanism and without locking mechanism for navigation.

Movie S7 (.mp4 format). Navigation demonstration in a synthetic stomach model.
